# [^18^F]FDG PET imaging in the differentiation of cardiac masses: an updated systematic review and dual Meta-Analysis of diagnostic performance and parameter variability

**DOI:** 10.1007/s00259-025-07289-w

**Published:** 2025-04-29

**Authors:** Alessio Rizzo, Vincenzo Viccaro, Anna Giulia Pavon, Laura Anna Leo, Giorgio Treglia

**Affiliations:** 1 Nuclear Medicine, Candiolo Cancer Institute, FPO–IRCCS, Turin, Italy; 2 Division of Cardiac Imaging, Istituto Cardiocentro Ticino, Ente Ospedaliero Cantonale, Lugano, Switzerland; 3 Faculty of Biomedical Sciences, Università della Svizzera Italiana, Lugano, Switzerland; 4 Faculty of Biology and Medicine, University of Lausanne, Lausanne, Switzerland; 5 Division of Nuclear Medicine, Imaging Institute of Southern Switzerland, Ente Ospedaliero Cantonale, Bellinzona, Switzerland

**Keywords:** Cardiac masses, [^18^F]FDG PET/CT, Diagnostic accuracy, Meta-analysis, Oncology

## Abstract

**Introduction:**

Cardiac masses (CMs) encompass a heterogeneous group of benign, malignant, and pseudotumoural lesions, posing diagnostic challenges due to their rarity and varied aetiologies. Given the limitations of conventional imaging modalities in differentiating benign from malignant masses, [^18^F]FDG PET/CT has emerged as a promising technique by providing metabolic information. This systematic review and meta-analysis aimed to evaluate the diagnostic performance of [^18^F]FDG PET/CT in characterising CMs and assess semi-quantitative parameters’ role in distinguishing malignant from benign lesions.

**Methods:**

A systematic review and meta-analysis were conducted, including studies evaluating the diagnostic accuracy of [^18^F]FDG PET/CT in CMs. Sensitivity and specificity were pooled using a random-effects model, and a secondary analysis examined differences in SUVmax between malignant and benign lesions.

**Results:**

Fifteen studies enrolling 1114 patients met inclusion criteria. The pooled sensitivity and specificity of [^18^F]FDG PET/CT in detecting malignant CMs were 89.2% (95% CI: 85–92%) and 82.8% (95% CI: 78–87%), respectively. Malignant lesions exhibited significantly higher SUVmax values (range: 5.6–14.3) than benign masses (range: 1.1–5.3, *p* < 0.001). PET/CT proved particularly effective in cases with inconclusive findings from echocardiography, cardiac magnetic resonance, or CT, contributing to biopsy guidance and treatment planning.

**Conclusions:**

[^18^F]FDG PET/CT demonstrates robust diagnostic accuracy in differentiating benign from malignant cardiac masses, with SUVmax as a valuable malignancy marker. Its integration into multimodal imaging strategies enhances diagnostic certainty and optimises patient management. Despite these advantages, standardised imaging protocols and further multicentre prospective studies are warranted to refine its clinical application and validate its prognostic potential.

**Supplementary Information:**

The online version contains supplementary material available at 10.1007/s00259-025-07289-w.

## Introduction

Cardiac masses (CMs) are a rare entity, although their prevalence has been increasing over the past decade due to the growing use of non-invasive techniques [1, 2]. The incidence is estimated between 0.001% and 0.3% in the general population [1]. These masses are broadly classified as benign, malignant, or pseudotumoral. CMs account for primary cardiac tumours (PCT) or secondary lesions. PCTs are typically benign (75-90%), with myxomas being the most common, followed by fibroelastomas and lipomas [3]. Among malignant PCTs, sarcomas are the most frequent (65-88%), followed by lymphoma (27%) and mesothelioma (8%) [3]. Secondary or metastatic cardiac tumours are significantly more common than PCTs, occurring 20 to 40 times more frequently [4]. Finally, a heterogeneous group of tumour-like masses that includes thrombi, cysts, lipomatosis, valvular nodules, Lambl’s excrescences, and other anatomical variants must also be considered when discussing CMs [5–7]. Given their heterogeneity, the prognosis, potential consequences, and treatment may vary, making a definitive diagnosis essential [8, 9]. The final diagnosis can always be achieved through endomyocardial biopsies (EMB), though this procedure is invasive, not always available, and limited by the possibility of sampling errors. Thanks to the recent advancement in cardiac imaging techniques [10], the multimodal imaging approach in clinical practice has proven effective in identifying red flags for malignancy, reducing diagnostic delays, optimising resources, and improving outcomes [1, 11]. Although there is no standardised approach, echocardiography is the first-line examination, detecting anatomical features suggestive of malignancy and excluding anatomical variants. Cardiac magnetic resonance (CMR) and computed tomography (CT) may be considered in the second line, as they characterise tissue composition and offer anatomical details. However, these methods bear significant limitations that may render CM interpretation inconclusive.

In this context, positron emission tomography/computed tomography (PET/CT) with 2-[^18^F]fluorodeoxyglucose ([^18^F]FDG) has emerged as a promising imaging modality, uniquely combining metabolic and anatomical information. [^18^F]FDG PET provides insights into tumour glucose metabolism, with semi-quantitative parameters such as maximum standardised uptake value (SUVmax), metabolic tumour volume (MTV), and total lesion glycolysis (TLG) demonstrating the potential for differentiating between benign and malignant CMs [10]. Although it has proven effective in a few studies, its use is currently limited by availability, the lack of large registries and costs.

This systematic review and meta-analysis aims to perform a qualitative and quantitative evaluation of the diagnostic performance of hybrid [^18^F]FDG PET, coregistered with CT or MR imaging in differentiating benign from malignant CMs. Additionally, it seeks to analyse differences in semi-quantitative parameters between these categories, thereby elucidating the role of hybrid [^18^F]FDG PET/CT imaging in the comprehensive diagnostic and therapeutic management of CMs.

## Materials and methods

### Protocol

This systematic review and meta-analysis was conducted following a preconceived protocol [12] and in accordance with the PRISMA 2020 statement [13]. The PRISMA checklist is included in *Supplementary Table 1*. The protocol was not registered in any database, such as PROSPERO.

The primary question addressed in this meta-analysis was: is [^18^F]FDG-PET effective in the management of patients with CMs for discriminating between benign and malignant lesions? The secondary question was: Are semi-quantitative PET parameters (e.g., SUVmax) valuable in discriminating malignant cardiac lesions from benign ones?

The study design follows the PICO framework:

- Population (P): Patients with detected CMs.
- Intervention (I): [^18^F]FDG PET imaging performed for lesion evaluation.
- Comparator (C): None explicitly included, as the analysis focuses on [^18^F]FDG PET performance.
- Outcome (O): Diagnostic accuracy of [^18^F]FDG PET in identifying malignant lesions confirmed through histological examination.

### Strategy for literature research and information sources

Two authors (A.R. and G.T.) independently conducted a comprehensive literature search using PubMed/Medline and the Cochrane Library databases. The following search string was employed:

A: “PET” OR “positron”; B: “cardiac masses” OR “intracardiac masses” OR “pericardial masses” OR “myocardial masses”. No language or publication year restrictions were applied, ensuring a broad search strategy. Additionally, the references of included articles were screened to identify further relevant studies. The final literature search update was performed on January 26, 2025.

### Eligibility criteria

The investigators incorporated clinical research papers yielding insights into using [^18^F]FDG PET in characterising CMs. Reviews, letters, editorials, case reports, case series, and original documents concerning different fields were excluded from the analysis. To ensure that only pertinent studies were included in the meta-analysis, the papers with insufficient data for pooling the sensitivity and specificity of [^18^F]FDG PET in characterising CMs, as well as separate mean values and standard deviations of SUVmax for benign and malignant lesions, were excluded from the analysis. Studies with potential overlap of patient data from other papers were also excluded.

### Data selection, collection and extraction

The titles and abstracts of the retrieved publications were evaluated based on predefined eligibility criteria. Final decisions regarding inclusion were made independently for both the systematic review and meta-analysis. To minimise bias, two researchers (A.R. and G.T.) independently collected and extracted data from the included studies. Extracted data encompassed study details (authors, country, year, methodology, and funding), patient information (sample size, gender, age, and additional tests), and specifics of the PET procedure (radiopharmaceuticals, imaging method, patient preparation, administered activity, and timing of image acquisition).

### Quality assessment (risk of bias assessment)

The QUADAS-2 tool, designed to evaluate the quality of research on diagnostic accuracy, was used to assess potential bias in individual studies and their relevance to the review question. Two authors (A.R. and G.T.) independently appraised the quality of each study included in the systematic review and meta-analysis. Four domains were examined for risk of bias: patient selection, index test, reference standard, and flow and timing. Additionally, three areas (patient selection, index test, and reference standard) were evaluated for applicability.

### Effects metrics

The primary outcomes of the meta-analysis were the sensitivity and specificity of [^18^F]FDG PET in discriminating malignant CMs from benign ones. Moreover, the authors performed a secondary analysis to establish if there were significant differences in mean SUVmax values between benign and malignant cardiac lesions.

### Statistical analysis

Pooled sensitivity and specificity of [^18^F]FDG PET in identifying malignant CMs were calculated using the DerSimonian and Laird random-effects model, with results presented as 95% confidence intervals (C.I.s) and illustrated through forest plots. Statistical heterogeneity was assessed using the I² index, with values over 50% indicating significant heterogeneity. A secondary analysis was also performed to examine differences in mean SUVmax values between benign and malignant cardiac lesions. All calculations were performed using OpenMeta[Analyst]^®^, supported by the Agency for Healthcare Research and Quality (AHRQ), Rockville, MD, USA.2.9. Additional Analyses.

If necessary, subgroup analyses were performed after identifying statistically significant heterogeneity within the encompassed studies, considering study design, patient characteristics, technical factors, and the clinical contexts under investigation.

## Results

### Literature search and study selection

**Fig. 1:**
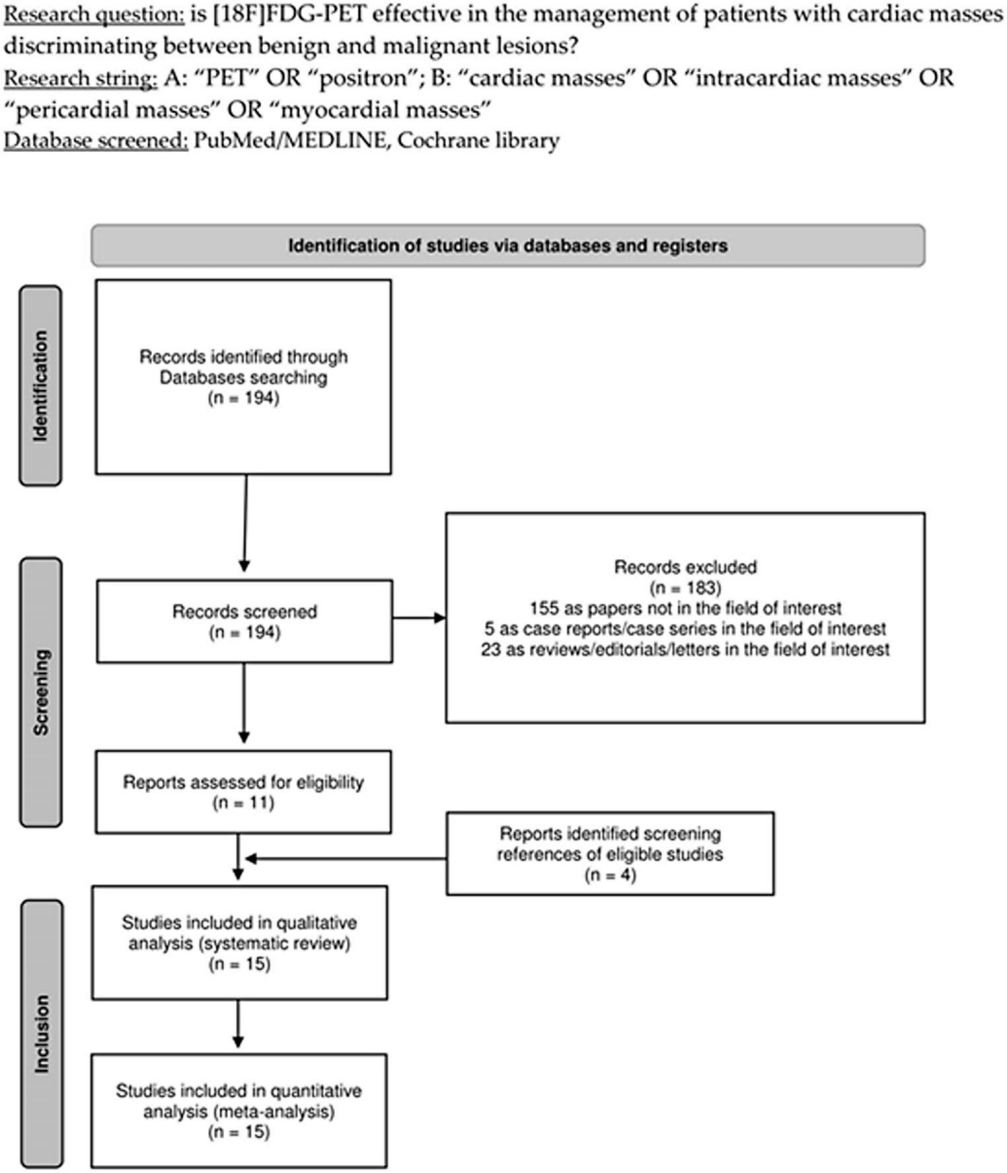
Flow diagram of the study selection process

The comprehensive literature review retrieved 194 records. In accordance with the details provided in the materials and methods section, these publications were evaluated for eligibility based on predetermined inclusion and exclusion criteria, resulting in the rejection of 183 documents (comprising 23 reviews/editorials/letters relevant to the field, 5 case reports pertinent to the field, and 155 articles unrelated to the topic of interest). The eleven remaining papers were deemed appropriate for inclusion in the systematic review (qualitative synthesis) and meta-analysis (quantitative synthesis) [14–24]. Four additional research articles that met the inclusion criteria were identified upon evaluating the references of these articles. Figure 1 delineates the research selection process [25–28].

### Study characteristics

The 15 papers included in the present systematic review and meta-analysis enrolled 1114 patients. These studies were conducted across multiple countries, with the most extensive contributions from China (6 studies), followed by the United States (3 studies), Germany (2 studies), France (2 studies), Italy (1 study), and Japan (1 study). One included study was prospective [24], while the remaining 14 were retrospective [14–23, 25–28]. Regarding study settings, 13 studies were conducted in a single centre [14–22, 24, 26–28], whereas two involved multiple centres [23, 25]. Only two studies disclosed external funding sources [14, 23]. Table 1 synthesises the general study information.

**Table 1:** General study information, patient key characteristics and clinical settings

Authors [Ref.]	Year	Country	Study design/number of involved centres	Sample size (no. patients)	Mean/median age (Years)	Gender (male %)	Gold standard	Funding sources
Dan Shao et al. [27]	2011	China	Retrospective/ Single centre	23	Mean: 55	61%	Histopathology	None declared
Rahbar et al. [26]	2012	Germany	Retrospective / Single centre	24	Mean: 59	46%	Histopathology	None declared
Kikuchi et al. [28]	2013	Japan	Retrospective / Single centre	17	Mean: 55	53%	Histopathology	None declared
Nensa et al. [24]	2015	Germany	Prospective / Single centre	20	Mean: 58	35%	Histopathology	None declared
Chan et al. [23]	2019	China&U.S.A.	Retrospective / Multicentric	121	Mean: 54	63%	Histopathology / Follow-up	National Institutes of Health grant
D’Angelo et al. [20]	2020	Italy	Retrospective / Single centre	60	Mean: 59	62%	Histopathology	None declared
Lemasle et al. [21]	2020	France	Retrospective / Single centre	112	Mean: 58	70%	Histopathology	None declared
Liu et al. [19]	2020	China	Retrospective / Single centre	46	Mean: 44.8	54%	Histopathology	None declared
Qin et al. [22]	2020	China	Retrospective / Single Centre	64	Mean: 51.2	53%	Histopathology / Follow-up	None declared
Meng et al. [25]	2021	China	Retrospective / Multicentric	38	Mean: 49.5	53%	Histopathology	None declared
Aghayev et al. [17]	2022	U.S.A.	Retrospective / Single centre	72	Median: 63	53%	Histopathology	None declared
Yin et al. [18]	2022	China	Retrospective / Single centre	59	Mean: 50	54%	Histopathology	None declared
Mikail et al. [16]	2022	France	Retrospective / Single centre	28	Median: 60.5	39%	Histopathology	None declared
De la Fuente et al. [15]	2022	U.S.A.	Retrospective / Single centre	389	Mean: 59	46%	Histopathology	None declared
Hu et al. [14]	2024	China	Retrospective / Single centre	41	Mean: 49	54%	Histopathology	Guizhou Province science and technology plan project

The patients enrolled in the included studies ranged from 17 to 389. The average age of participants spanned from 44.8 to 63 years, and the proportion of male patients varied between 35% and 70%. All studies utilised histopathology as a benchmark for definitive diagnosis of CMs, while two studies additionally incorporated follow-up data to establish the final diagnosis [22, 23]. A thorough analysis of enrolled patient data is available in Table 1.

All included studies employed [^18^F]FDG for PET execution. Fourteen studies used PET/CT as the hybrid imaging modality [14–23, 25–28], while one used PET/MR [24]. A carbohydrate-free diet to suppress physiological myocardial activity was adopted in 8 studies [19, 20, 24–28], while the remaining studies either did not use this preparation or did not report it [14–18, 21, 23]. The administered activity ranged from 3.7 to 7.4 MBq/kg for body-weight-based dosing and between 199 and 543 MBq when reported as an absolute value. The uptake time for imaging varied across studies, ranging from 55 to 94 min, with most studies standardising an interval of 60 min. Image analysis was performed using both qualitative and semi-quantitative approaches in most studies, with semi-quantitative measures such as SUVmax reported in all studies but two [15, 21]. About half of the studies employed additional metrics, such as target-to-background ratio TBR, MTV, and TLG [14, 16, 18–20, 22, 25, 26]. For studies measuring TBR, the mediastinal blood pool was consistently used as the reference background. Table 2 synthesises the data about index test key characteristics.

**Table 2:** Index test key characteristics

Authors [Ref.]	Tracer	Hybrid imaging	Carbohydrate-free diet before exam	Tomograph	Administered activity	Uptake time (minutes)	Image analysis
Dan Shao [27]	[^18^F]FDG	PET/CT	Yes	Sensation Biograph Somatom 16 (Siemens)	3.7–7.4 MBq/kg	60	Qualitative, Semiquantitative (SUVmax)
Rahbar et al. [26]	[^18^F]FDG	PET/CT	Yes	Biograph Sensation 16 (Siemens)	5 MBq/kg	68	Qualitative, Semiquantitative (SUVmax), TBR
Kikuchi et al. [28]	[^18^F]FDG	PET/CT	Yes	Biograph 64 (Siemens)	Not avaialble	60	Qualitative, Semiquantitative (SUVmax)
Nensa et al. [24]	[^18^F]FDG	PET/MR	Yes	Biograph MR (Siemens)	199 ± 58 MBq	94	Qualitative, Semiquantitative (SUVmax)
Chan et al. [23]	[^18^F]FDG	PET/CT	Not reported	Discovery (GE)	459 ± 33 MBq	60	Qualitative, Semiquantitative (SUVmax)
D’angelo et al. [20]	[^18^F]FDG	PET/CT	Yes	Not available	Not available	60	Qualitative, Semiquantitative (SUVmax, MTV, TLG)
Lemasle et al. [21]	[^18^F]FDG	PET/CT	Not reported	Not available	Not available	Not reported	Qualitative
Liu et al. [19]	[^18^F]FDG	PET/CT	Yes	Biograph HI-REZ 16 (Siemens)	5.55 MBq/kg	60	Qualitative, Semiquantitative (SUVmax, TBR)
Qin et al. [22]	[^18^F]FDG	PET/CT	Not reported	Discovery VCT (GE)	3.7–5.55 MBq/Kg	60	Qualitative, Semiquantitative (SUVmax, MTV, TLG)
Meng et al. [25]	[^18^F]FDG	PET/CT	Yes	Biograph mCT (Siemens)	254 MBq	60	Qualitative, Semiquantitative (SUVmax, TBR)
Aghayev et al. [17]	[^18^F]FDG	PET/CT	No	Discovery (GE)	543 MBq	60	Qualitative, Semiquantitative (SUVmax)
Yin et al. [18]	[^18^F]FDG	PET/CT	No	Discovery VCT 64 (GE); uMI150 (United Imaging)	3.7–4.4 MBq/kg	60	Qualitative, Semiquantitative (SUVmax, TBR)
Mikail et al. [16]	[^18^F]FDG	PET/CT	Not reported	Discovery 690 (GE)	4 MBq/kg	60	Qualitative, Semiquantitative (SUVmax, TBR)
De la Fuente et al. [15]	[^18^F]FDG	PET/CT	Not reported	Not reported	Not reported	Not reported	Not reported
Hu et al. [14]	[^18^F]FDG	PET/CT	Not reported	Biograph mCT (Siemens)	3.7–5.5 MBq/Kg	55–65	Qualitative, Semiquantitative (SUVmax, MTV TLG TBR)

Legend: CT: computed tomography, FDG: fluorodeoxyglucose, MR: magnetic resonance; MTV: metabolic tumour volume; PET: positron emission tomography; SUV: standardised uptake value; TBR: target-to-background ratio; TLG: total lesion glycolysis

### Risk of bias and applicability

**Fig. 2:**
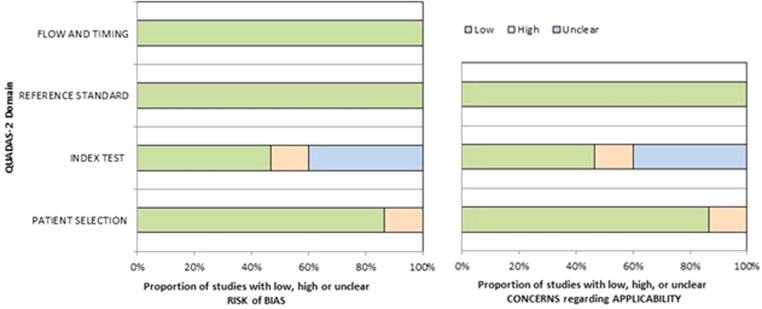
A graphical overview of the quality assessment performed employing the QUADAS-2 tool. The reviewers categorised the studies included in the systematic review based on their level of bias or applicability issues for specific topics stated on the ordinate axis. The abscissa represents the percentage of studies. According to the graph, about 40% of included studies present a risk of bias in the “index test” domain due to the absence of a clear statement about patient preparation before the PET exam, an essential step toward making a precise and reproducible study

The thorough assessment of the risk of bias and concerns about the applicability of the included studies according to QUADAS-2 is furnished in Fig. 2.

### Results of individual studies (qualitative synthesis)

Based on the data available in the included studies, a total of 429 malignant lesions and 264 benign lesions were reported [14–28]. The average SUVmax (reported in terms of mean or median) of malignant lesions ranged from 5.6 to 14.3, while for benign lesions, it ranged from 1.1 to 5.3 [14, 16–28].

The included studies consistently reported high diagnostic sensitivity and specificity of [^18^F]FDG PET in detecting malignant CMs [14–28]. Compared to traditional imaging modalities like TTE, PET demonstrated superior ability in identifying malignancies and refining diagnostic accuracy. For instance, studies by Yin et al. and Aghayev et al. highlighted cases where [^18^F]FDG PET altered the initial diagnostic impression provided by other modalities, confirming its pivotal role in improving the overall diagnostic workflow [17, 18]. Specifically, Yin et al. observed that [¹⁸F]FDG PET/CT modified the initial transthoracic echocardiography diagnosis in 15 patients (25.4%), accurately identified two malignant pericardial masses overlooked by transthoracic echocardiography, and recognised extracardiac metastatic lesions in 7 of 29 patients (24.1%) with malignant cardiac tumours [18]. Moreover, in the study by Aghayev et al., FDG-PET/CT helped reclassify cases that appeared benign on CMR. One patient had a cardiac fibroadipose mass with features suggestive of malignancy on CMR, but only minimal FDG uptake, leading to a final benign diagnosis. Conversely, another patient had a mass with few malignant features on CMR, but intense FDG uptake and subsequent confirmation of malignancy. These examples show that PET can both upstage and downstage CMR-based impressions, directly impacting clinical decision-making [17]. This enhanced sensitivity makes [^18^F]FDG PET particularly valuable in scenarios where malignancy is suspected but not confirmed by other imaging.

A wide range of aetiologies for both benign and malignant cardiac lesions were reported in the included papers [14–28]. Among the benign lesions, the most commonly identified were myxomas, followed by lipomas, fibroelastomas, hemangiomas, and thrombi. Less frequent benign entities included paragangliomas, granular cell tumours, and caseous mitral annulus calcifications. For malignant lesions, the predominant aetiologies included angiosarcomas, leiomyosarcomas, and lymphomas, along with metastatic tumours originating from various primary cancers, including lung cancer, melanoma, sarcomas, renal cell carcinoma, and breast cancer. Other malignant types reported included synovial sarcomas, fibrosarcomas, and rhabdomyosarcomas.

The included studies highlighted the utility of [^18^F]FDG-PET in the comprehensive management of CMs [14–28]. Beyond differentiating between benign and malignant lesions, [^18^F]FDG PET was crucial in guiding therapeutic decisions and planning biopsies in complex cases. The imaging modality was particularly valuable in identifying metabolically active tumour regions suitable for targeted biopsy, thereby minimising procedural risks. Additionally, [^18^F]FDG PET helped detect extracardiac metastatic lesions, significantly influencing treatment strategies, especially in patients with advanced malignancies [16, 18, 22, 27].

The added value of [^18^F]FDGPET-based imaging was not only limited to diagnostic information. Indeed, several studies demonstrated the prognostic significance of semi-quantitative parameters in patients diagnosed with CMs. Patients with lesions exhibiting a high SUVmax were often associated with worse outcomes and shorter survival times. As highlighted by Qin et al., SUVmax values could stratify patients based on their risk of aggressive disease progression. This observation underscores PET’s dual role in providing accurate diagnostic information and insights into the potential clinical trajectory of patients [14, 22].

Concerning the comparison between [^18^F]FDG PET/CT and CMR, the available literature highlighted the complementary roles of PET- and CMR-based imaging in evaluating CMs. [^18^F]FDG PET proved particularly effective in assessing the metabolic activity of lesions, aiding in the differentiation between benign and malignant masses through semi-quantitative parameters such as SUVmax, TBR, and MTV. On the other hand, CMR provided superior anatomical and functional details, including tissue characterisation, vascularity, and myocardial involvement [16, 17, 21, 23, 24]. While PET offered incremental diagnostic value for identifying malignancies and staging metastatic lesions, some of the included studies observed that combining [^18^F]FDG PET and CMR did not significantly increase diagnostic accuracy compared to [^18^F]FDG PET alone. In this regard, Mikail et al., observed that the combination of PET and CMR yielded a sensitivity of 92.9% and specificity of 100%, while CMR alone achieved a specificity of 100% but slightly lower sensitivity (86.7%). This indicates that PET added sensitivity but not a substantial improvement in overall diagnostic accuracy [16]. Furthermore, Aghayev et al. showed that the combination of [¹⁸F]FDG PET/CT with CMR yielded a sensitivity of 85% and specificity of 88%, identical to those obtained with PET/CT alone [17]. However, integrating the two modalities has been suggested to enhance confidence in complex or borderline cases [16, 17, 23]. Table 3 synthesises the primary outcomes of the included studies.

**Table 3:** Outcomes of the included studies

Authors [Ref.]	Aim of the study	Number of malignant lesions	Average malignant Lesions SUV_max_	Diagnosis of malignant lesions	Number of benign lesions	Average benign Lesions SUV_max_	Diagnosis of benign lesions	Outcome
Dan Shao et al. [27]	Discriminating malignant and benign heat lesions	13	Median: 6.5	Sarcoma Pericardial Involvement of Lymphoma Pericardial mesothelioma Metastatic tumour	10	Median: 1.5	Thrombus Lipoma Myxoma Pericardial tuberculosis Pericarditis	Lesions’ SUVmax can predict the pathological status of cardiac and pericardial masses.
Rahbar et al. [26]	Discriminating malignant and benign heat lesions	17	Mean: 9.5±4.0	Angiosarcoma Liposarcoma Lymphoma Thymoma Metastatic tumour	7	Mean: 2.8±0.6	Fibroma Myxoma Hematoma Hemangioma Papillary fibroelastoma	FDG PET/CT provides incremental diagnostic information in determination of primary and metastatic heart malignancies
Kikuchi et al.* [28]	Investigating specific imaging findings in cardiac dominant DLBCL in comparison with other cardiac tumours	14	Mean: 14.3±8.4	DLBCL Sarcoma Metastatic tumour	3	Mean: 3.4	Fibroma Lipoma Benign granular cell tumour	FDG PET/CT, in combination with contrast-enhanced CT, was useful for distinguishing cardiac dominant DLBCLs,
Nensa et al. [24]	Assess FDG PET/MR ability to improve non-invasive diagnosis of CMs	7	Mean: 13.2±6.2	Ewing sarcoma Angiosarcoma Burkitt lymphoma Metastatic tumour	12	Mean: 2.3±1.2	Myxoma Fibroelastoma CCMA Thrombus Scar tissue after surgery	In selected patients, FDG PET/MR can improve non-invasive diagnosis and follow-up of CMs.
Chan et al.** [23]	Assess differential detection of CMs by CMR and FDG PET affects their relative utility for the management of CMs	46	Mean: 8.2 ±6.5	Lymphoma Sarcoma Metastatic tumour	20	Mean: 1.8±0.5	Thrombus	Both PET/CT and CMR showed good performance in differentiating malignant and benign lesions. Moreover, they deemed the prognosis in oncologic patients.
D’angelo et al. [20]	Assess if cardiac CT and FDG PET/CT may increase the diagnostic accuracy in CMs	40	Mean: 12.6±7.7	Sarcoma Lymphoma Metastatic tumour	20	Mean: 2.8±1.2	Myxoma Paraganglioma Thrombus Lipomatosis Pericardial cyst	FDG PET/CT provide valuable information for the identification of CMs.
Lemasle et al. [21]	Evaluate the performance and the decision-making impact of multimodal imaging, including CT, CMR and FDG PET/CT	78	Not reported	Primary tumour Metastatic tumour	34	Not reported	Benign primary tumour Thrombus Lipomatosis	All the studied cardiac imaging techniques are useful for CM exploration and management.
Liu et al. [19]	Evaluate the diagnostic accuracy of combined FDG PET/CT and contrast-enhanced CT in the differential diagnosis of primary cardiac tumours	17	Mean: 6.7±4.2	Angiosarcoma Leiomyosarcoma Lymphoma Metastatic tumour	29	2.7±0.4	Myxoma Hemangioma Lipoma	The use of both combined CT and PET/CT improved diagnostic accuracy in detecting malignant lesions.
Qin et al. [22]	Assess if FDG PET/CT in patients with CMs may provide useful information on diagnosis and prognosis	38	Mean: 11.6±5.1	Angiosarcoma Myofibroblastic sarcoma Synovial sarcoma Malignant peripheral nerve sheath tumour	27	Mean: 5.3±2.6	Myxoma Lipoma Leyommyomatosis Hemangioma Fibroelastoma Thrombus Valvular lesion	FDG PET/CT was valuable for diagnosis and prognostic assessment in patients with CMs.
Meng et al. [25]	Evaluate the diagnostic value of FDG PET/CT in determining CMs.	24	Mean: 11.9±7.1	Leiomyosarcoma Angiosarcoma Synovial sarcoma Fibrosarcoma Rhabdomyosarcoma Lymphoma Metastatic tumour	14	Mean: 2.35±1.3	Lipoma Neurolemmoma Elastofibroma Myxoma Hemangioma	FDG PET/CT accurately determined whether the cardiac lesions were benign or malignant.
Aghayev et al.*** [17]	Evaluate the role of CMR and FDG PET/CT in discriminating benign and malignant CMs	47	Median: 5.6	Spindle cell sarcoma Paraganglioma Angiosarcoma Synovial cell sarcoma Metastatic tumour	25	Median: 1.1	Thrombus Fibroadipose tissue CAT Lipomatosis Paraganglioma Myxoma	FDG PET/CT showed optimal diagnostic accuracy in differentiating benign and malignant CMs. Combining PET/CT and CMR did not increase the diagnostic accuracy.
Yin et al. [18]	Evaluate the ability of FDG PET/CT in cardiac/pericardial masses previously detected by ultrasound.	29	Mean: 9.3±6.5	Angiosarcoma Epithelioid sarcoma-like hemangioendothelioma Intimal sarcoma Malignant paraganglioma Pleomorphic leiomyosarcoma Lymphoma Thymoma Metastatic tumour	30	Mean: 2.5±1.0	Myxoma Thrombus Leiomyoma Inflammatory myofibroblastic tumour Hemangioma Amartoma	FDG PET/CT can improve the diagnostic work-up of CMs to determine if they are malignant.
Mikail et al. [16]	Test diagnostic performance of FDG PET/CT and CMR in characterising CMs	16	Median: 8.6	Angiosarcoma Synovial sarcoma Lymphoma Malignant paraganglioma Metastatic tumour	12	Median: 2.4	Septal tuberculosis granuloma Thrombus	Combining CMR with FDG PET/CT performs better than combining CMR and CT for cardiac tumour characterisation and staging.
De la Fuente et al.**** [15]	To define demographics and characteristics of patients and the accuracy of diagnosis with various imaging methods, including FDG PET/CT	18	Not reported	Primary malignant Metastatic tumour	3	Not reported	Myxoma Fibroelastoma	Since only a constrained subset of patients was submitted to PET/CT, no conclusions on its employment were drawn.
Hu et al. [14]	Explore the diagnostic value of FDG PET/CT metabolic parameters in the characterisation of CMs	25	Mean: 13.31±8.99	Angiosarcoma Myxomatodes sarcoma Rhabdomyosarcoma Lymphoma Mesothelioma Metastatic tumour	18	Mean: 3.35±2.46	Myxoma Hemangioma Pericarditis Thrombus Ventricular aneurism angioleiomyolipoma	FDG PET/CT metabolic parameters can discriminate benign and malignant CMs semi-quantitatively.

Legend: CAT: left atrium intracavitary calcified amorphous tumour; CCMA: caseous calcification of mitral annulus; CM: cardiac mass; CMR: cardiac magnetic resonance; CT: computed tomography; DLBCL: diffuse large B cell lymphoma; FDG: fluorodeoxyglucose; n.a.: not available: PET: positron emission tomography; SUV: standard uptake value

*: SUVmax was reported for only 12 cardiac lesions

**: 55 patients had no CMs and were used as a matched-paired control group

***: the median values are expressed as the ratio between the lesion’s SUVmax and background blood pool activity

****: of the 389 enrolled patients, only a subset of 21 patients was submitted to PET/CT in its diagnostic work-up

### Meta-analysis (quantitative synthesis)

The meta-analysis was split into two sub-analyses that examined the pooled sensitivity and specificity in determining the malignant nature of the identified CMs. Furthermore, a secondary analysis on a subset of studies was performed to assess if there is a significant difference in terms of mean SUVmax between benign and malignant lesions.

#### Sensitivity and specificity in assessing malignant cardiac masses

**Fig. 3:**
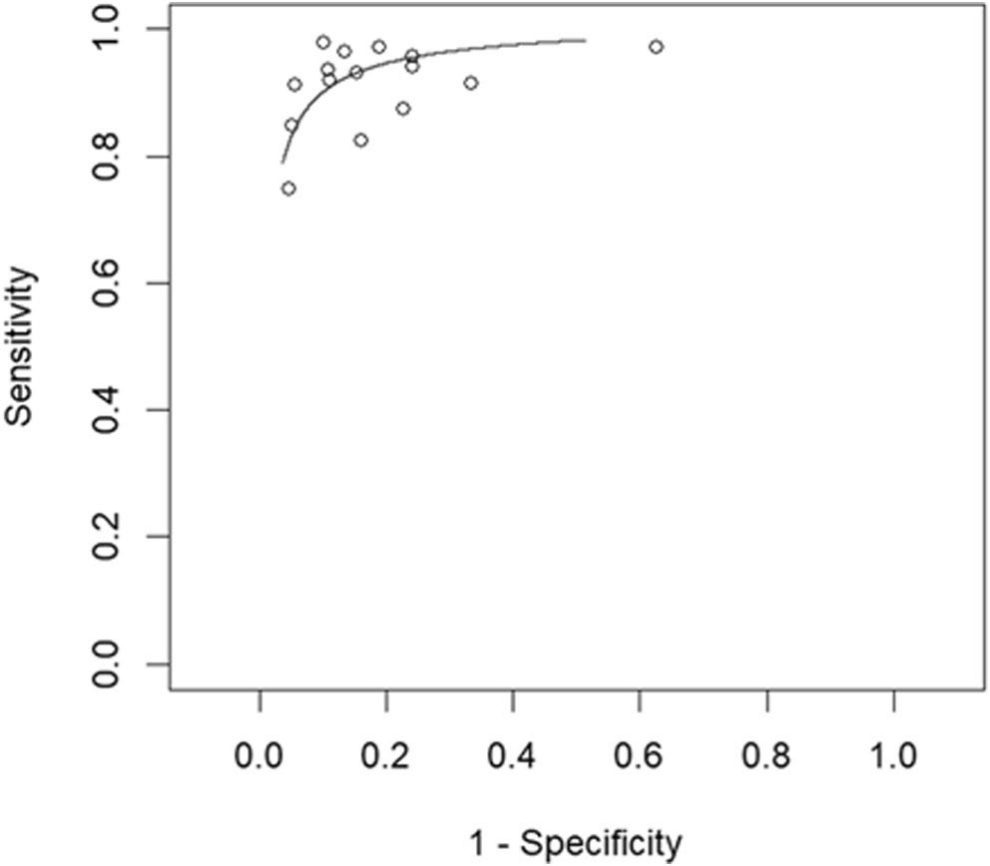
SROC curve of index test’s diagnostic accuracy

**Fig. 4:**
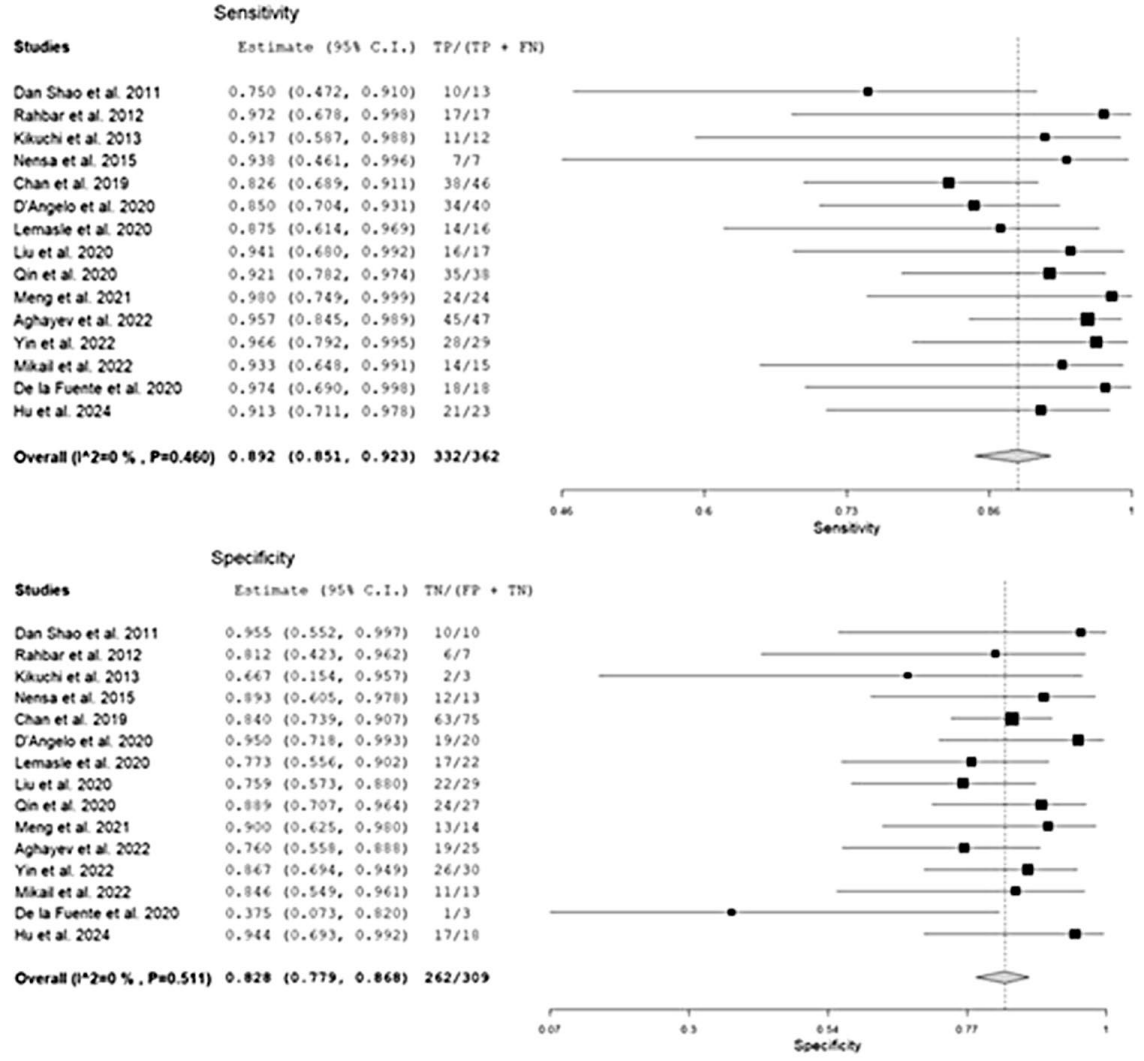
Pooled sensitivity and specificity of the index test in assessing CMs malignancies and relative forest plots. Legend: 95% C.I.: 95% confidence interval; TP: true positive; TN: true negative; FP: false positive; FN: false negative

Fifteen papers reporting the diagnostic accuracy of [^18^F]FDG PET in assessing CMs in 651 patients were included in the primary analysis [14–28]. Based on a per-lesion analysis, the pooled sensitivity and specificity of [^18^F]FDG PET in the determination of malignant CMs were 89.2% (95% CI: 85–92%) and 82.8% (95% CI: 78–87%), respectively. The relative summary receiver operating characteristics (SROC) and forest plots are shown in Figs. 3 and 4, respectively.

**Fig. 5:**
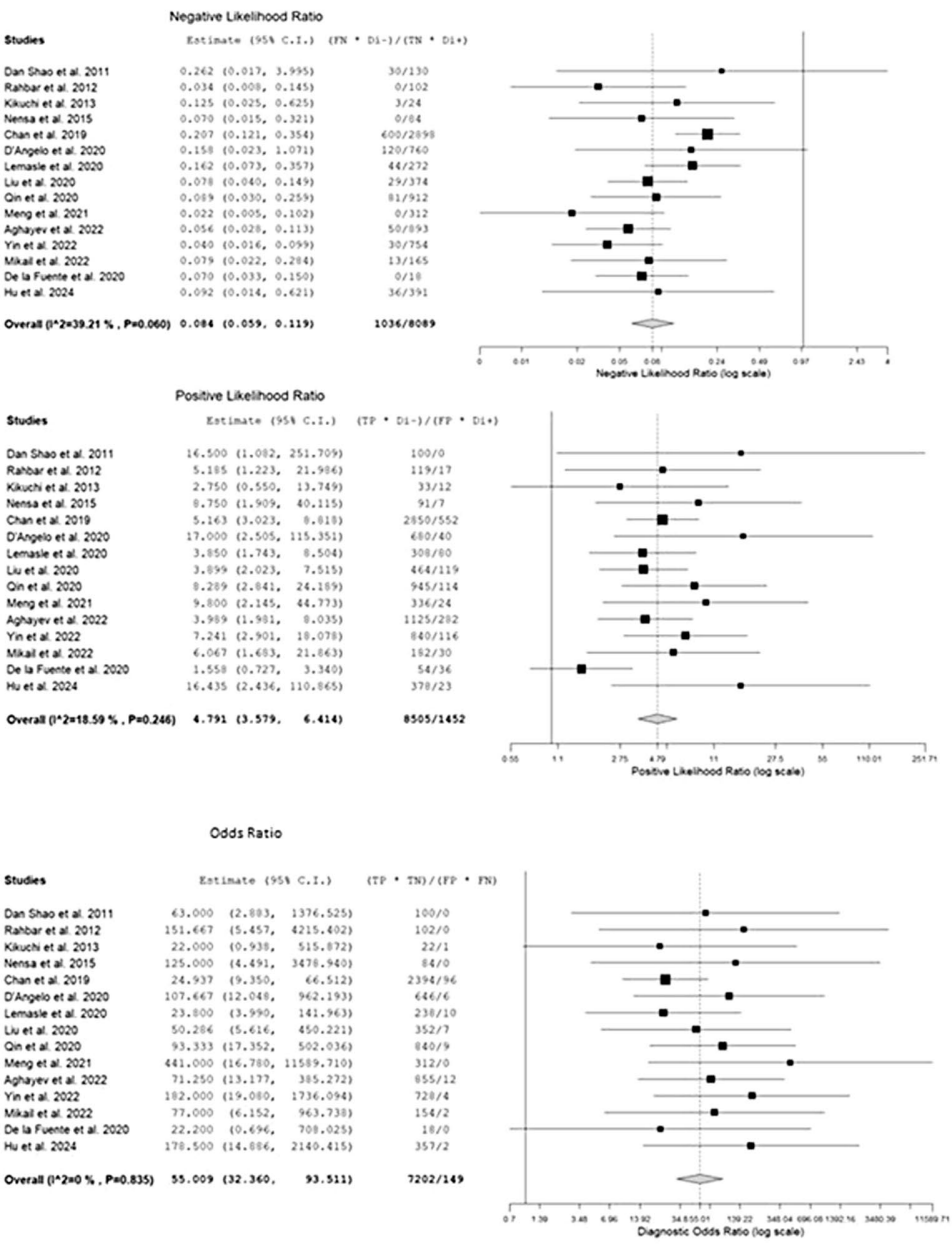
The index test’s negative and positive likelihood ratios and odds ratio with relative forest plots. Legend: 95% C.I.: 95% confidence interval; TP: true positive; TN: true negative; FP: false positive; FN: false negative

Figure 5 highlights the combined negative and positive likelihood ratios as well as the diagnostic odds ratio, which were, respectively, 0.08 (95% CI: 0.06–0.12), 4.79 (95% CI: 3.58–6.41), and 55.01 (95% CI: 32.36–93.51). Since the inconsistency index for the studies in this sub-analysis was continuously below 50%, there was no substantial statistical heterogeneity among them.

#### Differences in mean SUVmax between benign and malignant lesions

**Fig. 6:**
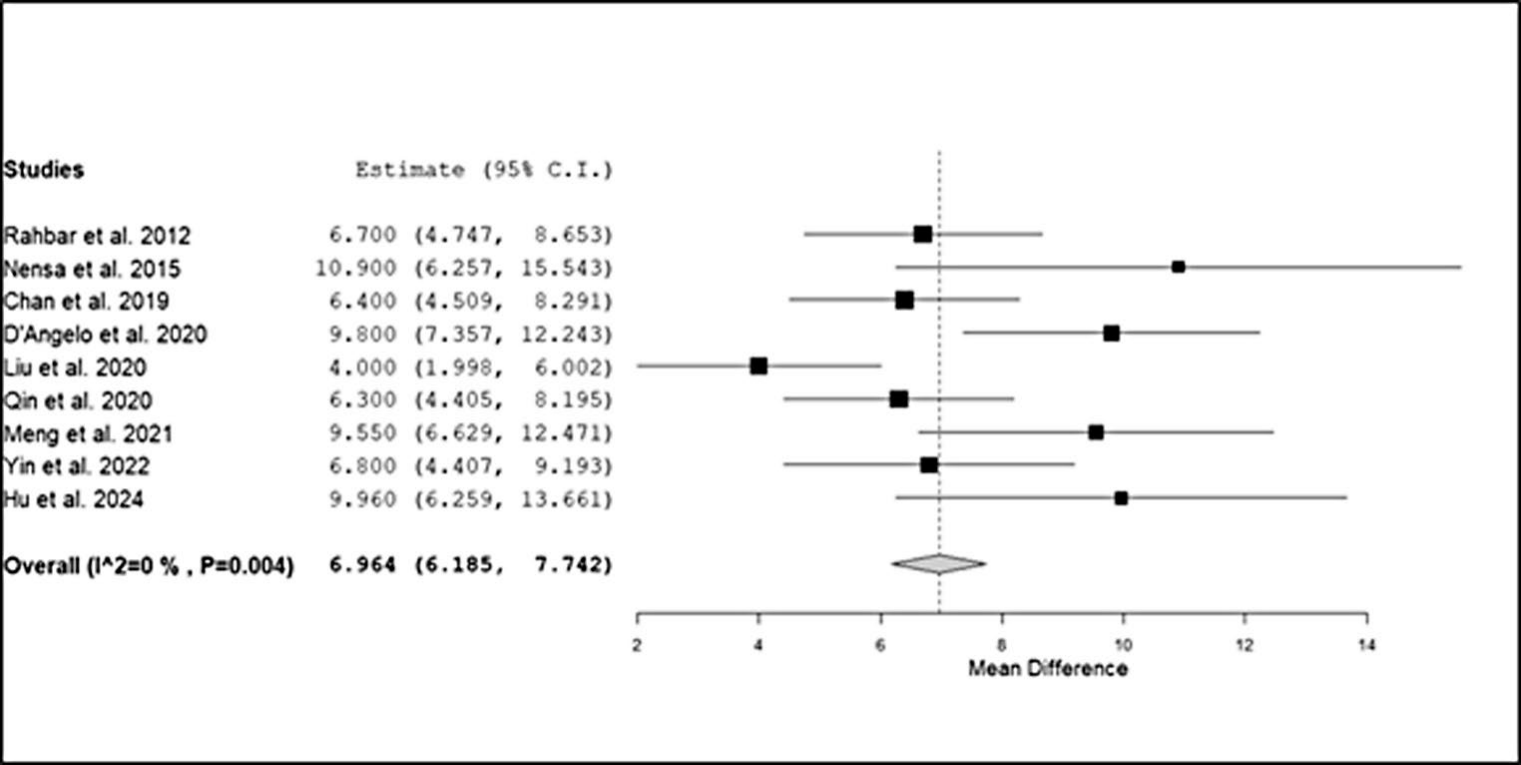
Forest plot of the mean difference analysis. Legend: 95% C.I.: 95% confidence interval

Nine papers reporting the mean value of SUVmax and relative standard deviation of malignant and benign CMs, enrolling 420 patients, were included in the secondary analysis [14, 18–20, 22–26]. This analysis revealed a significant difference in SUVmax between benign and malignant CMs (*p* < 0.001) with an I^2^ of 0%. The relative forest plot is reported in Fig. 6.

## Discussion

This meta-analysis has confirmed the pivotal role of [^18^F]FDG PET imaging in characterising CMs, taking into account an unprecedentedly large cohort and assessing semi-quantitative parameters for the first time. The main findings of our study are:

i) the high sensitivity and specificity of PET/CT and mean SUVmax in distinguishing benign from malignant CMs;
ii) the strong correlation between high SUVmax and poor prognosis.

### Multi-modality imaging approach: strengths and weaknesses

The multimodal imaging approach in characterising a CM’s benign or malignant nature has proven helpful in everyday clinical practice.

Transthoracic echocardiography remains the first-line diagnostic modality, providing high sensitivity for detecting intracardiac lesions due to its wide availability, low cost and non-invasive nature [10]. Red flags suggesting malignancy are: infiltration, polylobate mass, moderate-severe pericardial effusion, sessile attachment, inhomogeneity and righ-side localitation [29, 30]. In the case of small masses (< 1 cm), high mobility, or uncertain localisation, it is good practice to further investigate with a transesophageal echocardiogram [1]. Also, contrast-enhanced echocardiography might be useful in differentiating vascular tumours from thrombi [1, 31]. However, the accuracy of echocardiography is limited in cases of poor acoustic windows, challenges in tissue composition characterisation, or difficulties in assessing cardiac infiltration and extracardiac extension [29]​.

CMR is the gold standard non-invasive method for evaluating CMs. It has a higher spatial resolution than echocardiography and the distinctive advantage of tissue characterisation [11, 32–34]. Notable CMR limitations include: arrhythmias, long acquisition times (30 min to 1 h), hemodynamic instability, older intracardiac devices [35], claustrophobia and motion artefacts, slow flow and signal intensity variability in T2-weighted images [32]. Furthermore, CMR is not the most suitable technique for studying calcified or small and highly mobile valvular lesions.

Cardiac computed tomography (CT) is an alternative or a combined method to CMR. In addition to being the gold standard for calcified lesions and highly accurate for left atrial or appendage thrombi and masses involving the great vessels [36, 37], cardiac CT assists in surgical planning. Methodological weaknesses are also present, specifically radiation exposure, contrast-induced nephropathy or allergic side effects, and lower temporal resolution compared to CMR, although it is a quicker method. Nonetheless, these techniques cannot definitively distinguish benign from malignant lesions based solely on imaging features [10, 38].

In this context, hybrid [^18^F]FDG PET imaging, particularly [^18^F]FDG PET/CT, stands as a solid third-line technique thanks to the combination of metabolic and anatomical information. It effectively excludes malignancy, providing the highest sensitivity when the mass shows no radiotracer uptake. To enhance the visibility of neoplastic glucose-based metabolism, following a diet rich in fats for at least 12 h before the examination is essential, facilitating the transition of myocardial cells to lipid metabolism [20]. An exception is neuroendocrine tumours, whose metabolism is not based on glucose. In these cases, radiotracers such as [^68^Ga]Ga-DOTA-peptides binding somatostatin receptors and [^18^F]fluorodihydroxyphenylalanine ([^18^F]DOPA) should be employed for CM assessment [39]. Caution should be taken also for potential false positives: inflammatory diseases (such as sarcoidosis), endocarditis, or certain benign masses like myxomas and hemangiomas [1]. On the one hand, biopsy remains the gold standard for definitive diagnosis, and the [^18^F]FDG PET/CT can guide its execution towards the portion of the mass with the highest radiotracer uptake. On the other hand, the location of certain CMs can limit their accessibility. In such cases, PET/CT has proven to be a valuable and safe alternative method for distinguishing malignancy. However, there are histotypes, such as myxoid liposarcomas, that exhibit falsely benign metabolism [40]. A hybrid alternative to PET/CT is PET/MRI, which, in addition to lower radiation exposure, reduces false positives compared to PET/CT and standalone MRI thanks to its superior tissue and morphological characterisation [24, 41, 42]. However, its availability and cost limit its widespread use [24].

### Hybrid [^18^F]FDG PET imaging: a valuable tool in cardiac mass characterisation

This meta-analysis highlights the robust diagnostic performance of [^18^F]FDG PET in differentiating benign from malignant CMs. With pooled sensitivity and specificity of 89.2% and 82.8%, respectively, [^18^F]FDG PET demonstrates its great diagnostic accuracy [14–28]. These findings are further reinforced by the low heterogeneity observed across the included studies, underscoring the consistency and reliability of the diagnostic results [14, 18–20, 22–26].

The significant difference in mean SUVmax between benign and malignant lesions, ranging from 1.1 to 5.3 for benign lesions and 5.6 to 14.3 for malignant lesions, provides a valuable semi-quantitative parameter for clinical decision-making. This difference not only aids in distinguishing between benign and malignant masses but also offers prognostic insights. Studies included in this analysis, such as Qin et al., demonstrated that a higher SUVmax correlates with more aggressive disease behaviour and poorer outcomes, highlighting the dual diagnostic and prognostic utility of PET imaging [22].

Moreover, this review emphasises the complementary roles of [^18^F]FDG PET and other imaging modalities, particularly CMR. While PET excels in metabolic characterisation and identifying extracardiac metastases, CMR provides superior anatomical and functional details. However, studies such as those by Chan et al. and Aghayev et al. found no significant diagnostic advantage when combining PET and CMR, suggesting that PET alone is sufficient in most scenarios. Although CMR and PET are fundamentally different in principle, their findings often correlate strongly, especially in malignant lesions. In such cases, both modalities tend to reinforce the same diagnostic conclusion rather than contribute additive information. This redundancy reduces the theoretical benefit of combining the two. Furthermore, when discordant, the objective metabolic metrics from PET (e.g. SUVmax or TBR) often carry more clinical weight than morphological features alone, further limiting the practical gain from CMR in the presence of a conclusive PET result.Nevertheless, integrating the two modalities can enhance confidence in complex or borderline cases [17, 23].

An additional strength of [^18^F]FDG PET lies in its impact on patient management. Beyond its diagnostic capabilities, PET has proven invaluable in guiding therapeutic decisions, particularly in identifying metabolically active regions for targeted biopsy. This approach minimises procedural risks while ensuring accurate tissue sampling. Furthermore, the ability of PET to detect extracardiac metastatic lesions significantly influences treatment planning, especially in patients with advanced malignancies [17, 18, 22].

In 2022, Kim and colleagues published a meta-analysis investigating the role of [^18^F]FDG PET in assessing CMs. Both meta-analyses share several findings regarding the diagnostic accuracy of [^18^F]FDG PET for characterising CMs and underline the high sensitivity and specificity of metabolic imaging in distinguishing benign from malignant lesions [43]. However, there are notable differences in methodology and key outcomes. First, our meta-analysis encompassed 15 studies enrolling 1114 patients, providing a broader dataset than Kim et al. (6 studies with 408 patients). Moreover, our meta-analysis incorporates an analysis of SUVmax differences between benign and malignant lesions, demonstrating a significant range. Finally, the current meta-analysis accounts for a lower heterogeneity in sensitivity and specificity (I² consistently < 50%). In contrast, Kim et al. noted some heterogeneity due to differences in PET interpretation criteria and imaging protocols​; this last difference may be due to the constrained available literature when the meta-analysis was performed.

This meta-analysis aligns with the findings of Angeli et al. [10], which emphasise the role of [^18^F]FDG PET as a key imaging modality when CMR or CT results are inconclusive. Both analyses highlight PET’s strengths in metabolic characterisation through SUVmax, aiding the differentiation between benign and malignant masses and supporting systemic staging. Angeli et al. further stress PET’s utility in hybrid imaging (e.g., PET/MR) for reducing false positives and enhancing tissue characterisation [10, 24]. However, they also note limitations, such as challenges with physiological myocardial uptake and reduced specificity in inflammatory conditions, underscoring the need for rigorous dietary preparation. These findings reinforce [^18^F]FDG PET’s value within a multi-modality framework, particularly for complex cases, while highlighting the importance of standardised protocols and complementary imaging techniques.

### Cost-effectiveness and clinical applicability

The cost and limited availability of [¹⁸F]FDG PET/CT are recognised barriers to its widespread use, particularly in low- and middle-income countries. However, based on the results of this systematic review and meta-analysis, PET/CT may help reduce overall healthcare costs by preventing unnecessary invasive procedures (and related complicancies) and avoiding delays in diagnosis.

Several studies support this view. Yin et al. reported that PET/CT changed the initial diagnostic impression in over 25% of cases and detected extracardiac disease in 24% of malignant tumours, directly influencing treatment planning [18]. Similarly, Qin et al. showed that SUVmax was an independent predictor of survival, allowing for early risk stratification and tailored therapy [22]. These insights cannot be easily obtained with conventional imaging alone. Therefore, while PET/CT may not be appropriate as a first-line modality in all settings, its selective use in diagnostically challenging or high-risk cases may optimise resource allocation and improve outcomes.

### Clinical implication

**Fig. 7:**
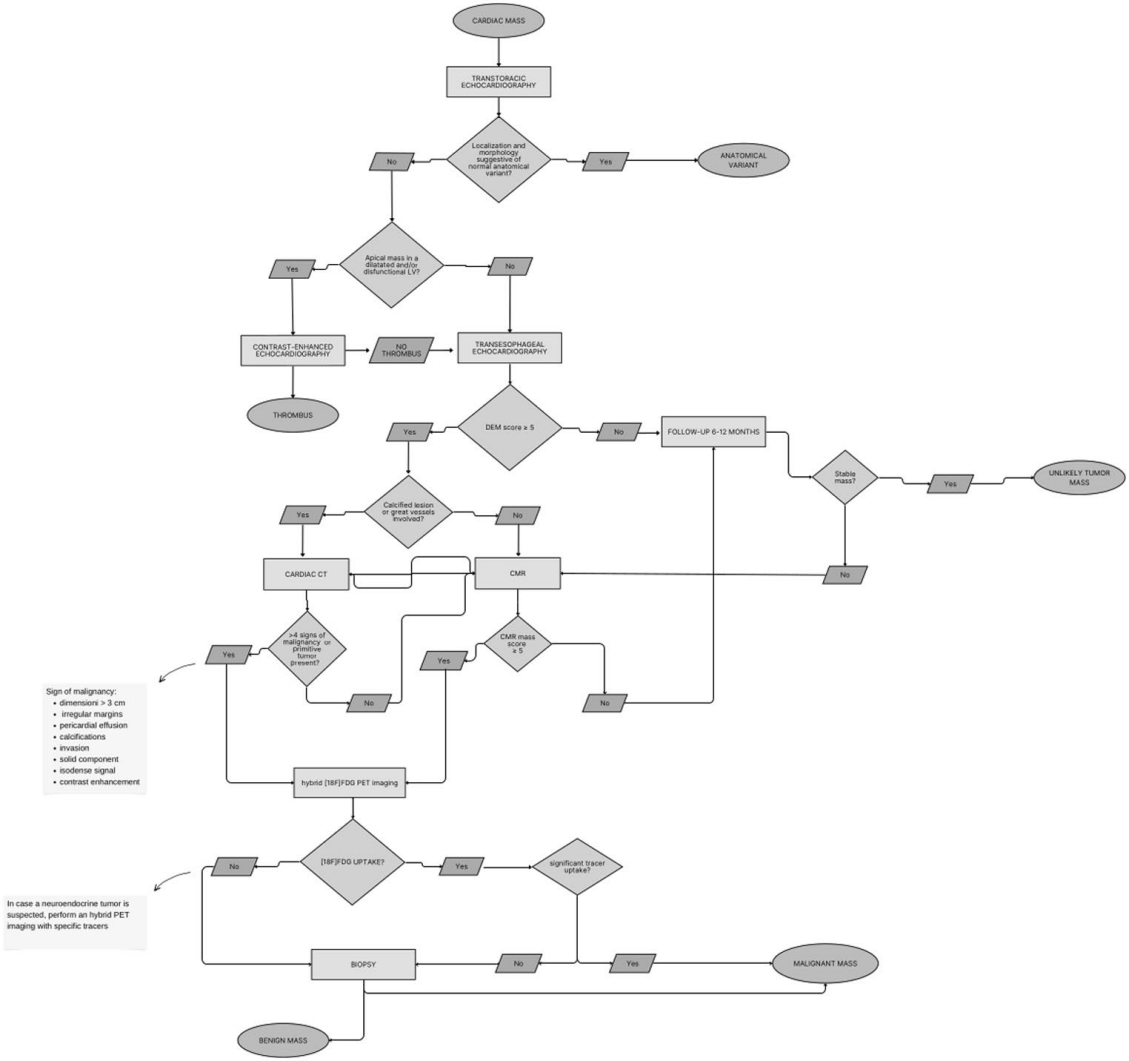
Proposed a diagnostic algorithm for optimal non-invasive assessment of CMs within a multimodal approach

Differentiating the nature of the mass has significant prognostic implications. Benign masses are associated with a favourable long-term prognosis [8], while malignant masses have a survival rate of 45.3% at 1 year and 11.5% at 5 years [8, 9]. Given the significant clinical implications of an accurate CM diagnosis and the lack of a standardised approach, we have proposed a diagnostic algorithm (Fig. 7) for optimal non-invasive methods within a multimodal approach.

### Limitations

Despite these findings, several limitations of the included studies warrant discussion. Most studies were retrospective in design, potentially introducing selection bias. Additionally, variability in patient preparation protocols, such as using carbohydrate-free diets to suppress physiological myocardial activity, might have influenced the reported SUVmax values. Future studies should aim to standardise imaging protocols to ensure greater reproducibility and comparability of results.

Lastly, while the evidence strongly supports the utility of [^18^F]FDG PET, its availability and cost may limit widespread use in specific healthcare settings. Therefore, incorporating PET into routine clinical workflows should be guided by the clinical context and availability of complementary imaging modalities.

## Conclusions

Hybrid [^18^F]FDG PET imaging demonstrates strong diagnostic and prognostic utility in evaluating CMs, especially in inconclusive echocardiography, CMR and/or cardiac CT. Its ability to characterise metabolic activity and guide management underscores its value as a cornerstone of this field. Further prospective, multicentric studies are needed to validate these findings and optimise the integration of PET into routine clinical workflows.

## Electronic supplementary material

Below is the link to the electronic supplementary material.

Supplementary Material 1

## Data Availability

The datasets generated during and/or analysed during the current study are available from the corresponding author on reasonable request.
